# Biomarker to predict treatment response in patients with HTLV-1 associated myelopathy/Tropical spastic paraparesis (HAM/TSP)

**DOI:** 10.1186/1742-4690-12-S1-P26

**Published:** 2015-08-28

**Authors:** Keiko Tamaki, Jun Tsugawa, Mika Matsuo, Yoshihisa Yamano, Yoshio Tsuboi

**Affiliations:** 1 Department of Neurology, FukuokaUniversity, Japan; 2 Department of Rehabilitation, Fukuoka University, Japan; 3 Department of Rare Diseases Research, Institute of Medical Science, St. Marianna University School of Medicine, Japan

## 

There are only a few studies which evaluate the utility of biomarkers to predict treatment response in HTLV-1 associated myelopathy/Tropical spastic paraparesis (HAM/TSP). Therefore, we investigated biomarkers of cerebrospinal fluid (CSF) in patients with HAM/TSP treated with high-dose methylpredonisolone (SP). Eight patients with HAM/TSP, who admitted to our hospital from 2012 to 2014 for treatment of HAM/TSP, were enrolled in this study. Three patients were rapidly progressive types and five patients were slowly progressive types. We evaluated the treatment response with SP and interferon-alpha (INF-α). The CSF was collected before and after the SP treatment. As the candidates of biomarker, CXCL10, neopterin, cell count and anti-HTLV-1 antibody titer in the CSF were measured. The motor functions were evaluated using the Osame Motor Disability Score (OMDS), 10m walking time and 6 minutes walking distance. The pre-treatment CXCL10 levels in the CSF of patients with rapidly progressive types were higher compared to those with slowly progressive types and the treatment response was remarkably better in rapidly progressive types. Level of CXCL10 in the CSF before treatment is related to improvement rate of motor function after the SP treatment in all patients. These results indicate that CXCL10 level in the CSF may be a biomarker to predict treatment response with SP.

